# CBX4 plays a bidirectional role in transcriptional regulation and lung adenocarcinoma progression

**DOI:** 10.1038/s41419-024-06745-z

**Published:** 2024-05-30

**Authors:** Ran Zhao, Yanxuan Guo, Linlin Zhang, Zhiyong Huang, Xuanyuan Li, Bei Lan, Diansheng Zhong, Hao Chen, Chenghao Xuan

**Affiliations:** 1 The Province and Ministry Co-sponsored Collaborative Innovation Center for Medical Epigenetics; Department of Medical Oncology, Tianjin Medical University General Hospital; Department of Biochemistry and Molecular Biology, Tianjin Medical University, Tianjin, 300070 China; 2 Department of Medical Oncology, Tianjin Medical University General Hospital, Tianjin, 300052 China; 3 Haihe Laboratory of Cell Ecosystem, College of Life Sciences, Nankai University, Tianjin, 300071 China

**Keywords:** Gene silencing, Non-small-cell lung cancer

## Abstract

Lung adenocarcinoma (LUAD) remains a leading cause of cancer-related mortality worldwide. Understanding the dysregulated epigenetics governing LUAD progression is pivotal for identifying therapeutic targets. CBX4, a chromobox protein, is reported to be upregulated in LUAD. This study highlights the dual impact of CBX4 on LUAD proliferation and metastasis through a series of rigorous in vitro and in vivo experiments. Further investigation into the underlying mechanism through high-throughput ChIP-seq and RNA-seq reveals that CBX4 functions in promoting LUAD proliferation via upregulating *PHGDH* expression and subsequent serine biosynthesis, while concurrently suppressing LUAD metastasis by inhibiting *ZEB2* transcription. CBX4 facilitates *PHGDH* transcription through the interaction with GCN5, inducing heightened histone acetylation on the *PHGDH* promoter. Simultaneously, the inhibition of *ZEB2* transcription involves CBX4-mediated recruitment of canonical PRC1 (cPRC1), establishing H2K119ub on the *ZEB2* promoter. These findings underscore CBX4’s pivotal role as a regulator of LUAD progression, emphasizing its diverse transcriptional regulatory functions contingent upon interactions with specific epigenetic partners. Understanding the nuanced interplay between CBX4 and epigenetic factors sheds light on potential therapeutic avenues in LUAD.

## Introduction

According to the GLOBOCAN 2020 database, lung cancer stands as the second most frequently diagnosed cancer and remains the foremost cause of cancer-related deaths globally, with an estimated 2.20 million new cases and approximately 1.8 million fatalities [1]. It manifests primarily in two main types: small cell lung cancer (SCLC) and non-small cell lung cancer (NSCLC). Lung adenocarcinoma (LUAD) is the most prevalent subtype of NSCLC [2]. Despite advancements, LUAD exhibits an overall five-year survival rate of merely 15 percent due to late-stage diagnosis and the tumor’s resistance to conventional chemotherapeutic agents [3]. Identifying pivotal factors driving the progression of LUAD holds the promise of unveiling novel diagnostic and therapeutic targets, carrying substantial clinical implications.

The precise orchestration of gene expression stands as a fundamental aspect of cellular regulation, and epigenetic factors play a pivotal role in this intricate process. Polycomb group (PcG) proteins have garnered significant attention due to their roles in development, cellular differentiation, and the maintenance of cellular identity [4, 5]. Primarily forming two major complexes-PRC1 and PRC2-PcG proteins contribute extensively to the dynamic regulation of chromatin structure and gene expression. In mammals, PRC2 is composed of enhancer of zeste homolog (EZH2 or EZH1), suppressor of zeste 12 (SUZ12), embryonic ectoderm development (EED), and retinoblastoma suppressor associated protein (RbAp46/48) [4]. This complex catalyzes the methylation of H3K27 through its EZH2/EZH1 subunit [4]. PRC1 executes gene silencing through RING1A/1B-catalyzed monoubiquitination of histone H2A at lysine 119 (H2AK119ub1), and the induction of chromatin compaction, thereby establishing a repressive chromatin state [6]. PRC1 can be categorized into two main groups: canonical PRC1 (cPRC1) and non-canonical PRC1 (ncPRC1). cPRC1 consists of Ring1 proteins (RING1a or RING1b), PcG ring-finger proteins (PCGF1-PCGF6), polyhomeotic homolog proteins (PHC1-PHC3), and chromobox proteins (CBX2, CBX4, CBX6, CBX7, or CBX8) [6]. The CBX proteins utilize their chromodomain to recognize and bind to histone H3 trimethylated at lysine 27 (H3K27me3), a mark catalyzed by the PRC2 complex. This initial recognition facilitates the recruitment of PRC1 to chromatin, culminating in subsequent ubiquitination of H2A and a repressive chromatin architecture [7–9].

Epigenetic abnormalities are widely acknowledged as pivotal contributors to the initiation and progression of cancer. Dysregulation in histone modifications and the loss of DNA methylation intricately link to various human cancers [10]. Furthermore, the promising clinical and preclinical outcomes achieved through drugs targeting epigenetic factors underscore the central role of epigenetics in cancer, emphasizing the necessity to explore novel epigenetic elements as therapeutic targets [11]. EZH2, a significantly overexpressed oncogene across multiple cancer types, has emerged as a prominent target for cancer therapy [12]. Notably, in 2020, the FDA approved tazemetostat as the first EZH2 inhibitor for treating epithelioid sarcoma [13]. EZH2 was reported to promote LUAD progression via regulating VEGFA/AKT signaling [14], and function as a prognostic-related biomarker in LUAD correlating with cell cycle and immune infiltrates [15]. Given that CBX4 discerns EZH2-mediated H3K27me3 to recruit PRC1 to maintain transcription repression, investigating the role of CBX4 in LUAD progression holds promise for identifying new epigenetic targets in LUAD therapy.

In this study, we have uncovered a dual role for CBX4 within LUAD progression. Our findings demonstrate its intricate involvement in both the proliferation and metastasis of LUAD through a series of in vitro and in vivo experiments. Notably, CBX4 serves as a promoter of LUAD proliferation by upregulating *PHGDH* expression and subsequent serine biosynthesis, while concurrently acting as an inhibitor of LUAD metastasis through the suppression of *ZEB2* transcription. We delve deeper into CBX4’s mechanism of inhibiting *ZEB2* transcription by elucidating its recruitment of cPRC1, thereby establishing H2K119ub on the *ZEB2* promoter. Simultaneously, our investigations reveal CBX4’s facilitation of *PHGDH* transcription via interaction with GCN5, leading to increased histone acetylation on the *PHGDH* promoter. These findings underscore the multifaceted transcriptional regulatory functions of CBX4, contingent upon its interactions with distinct epigenetic factors, consequently shedding light on its pivotal regulatory role in regulating LUAD progression.

## Results

### CBX4 promotes the proliferation of LUAD cells in vitro

**Fig. 1:**
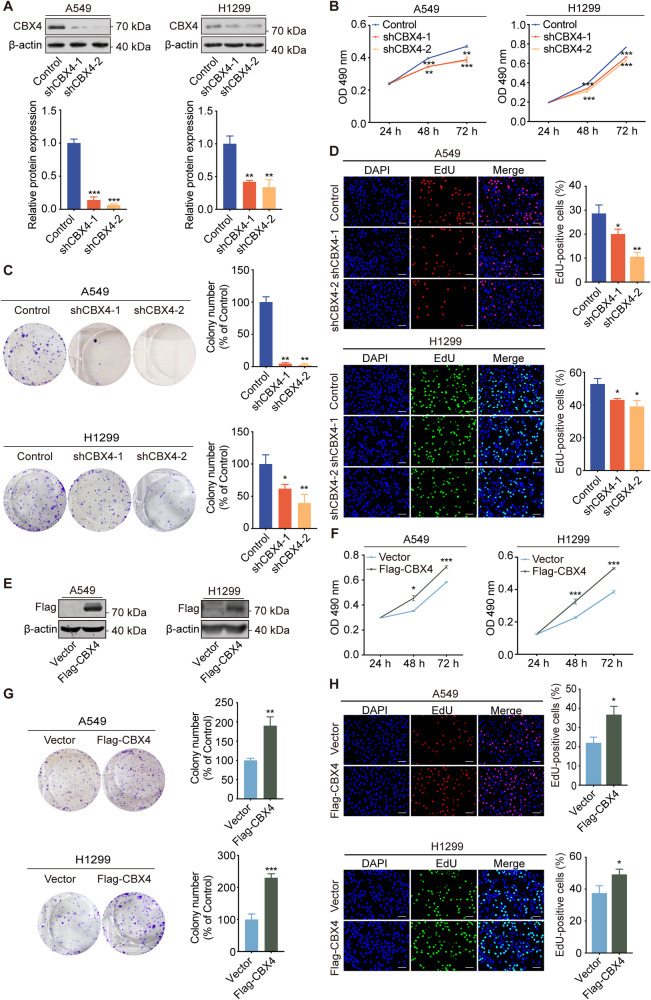
CBX4 promotes the proliferation of LUAD cells in vitro. **A** *CBX4* was knocked down by two independent short hairpin RNAs (shRNAs) in A549 and H1299 cells. Knockdown efficiency was assessed by Western blotting. The statistical results of the western blotting assays are presented in a bar chart. **B** MTT assays were performed in A549 and H1299 cells expressing control or *CBX4* shRNAs. **C** A549 and H1299 cells stably expressing control or *CBX4* shRNAs were cultured for 2 weeks, and stained with crystal violet. The number of colonies was counted. **D** A549 and H1299 cells expressing control or *CBX4* shRNAs were subjected to EdU‐incorporation assays, and the percentage of EdU‐positive cells was calculated. Scale bar, 100 μm. **E** Cell lysate from A549 and H1299 cells stably expressing empty vector or Flag-CBX4 was subjected to Western blotting using indicated antibodies. **F** MTT assays were conducted in A549 and H1299 cells with or without *CBX4* overexpression. **G** Colony formation assays were performed in indicated cells. **H** EdU-incorporation assays were conducted in A549 and H1299 cells with *CBX4* overexpression or not. Scale bar, 100 μm. In figures **A**–**D** and **F**–**H**, each bar represents the mean ± SD for *n* = 3; *P < 0.05, **P < 0.01, ***P < 0.001, indicating shCBX4 versus control, Flag-CBX4 versus vector (Student’s *t*-test).

CBX4 was reported by our previous study to be upregulated in LUAD [16]. To investigate the impact of *CBX4* knockdown on the proliferative capacity of LUAD cells, we employed lentiviruses expressing either control shRNAs or *CBX4* shRNAs to infect A549 and H1299 cells, establishing cell populations that stably expressed respective shRNAs. The efficiency of *CBX4* knockdown was confirmed through Western blot analysis (Fig. 1A). These *CBX4* knockdown and control cells were subsequently utilized for experiments pertaining to cell proliferation. Firstly, we performed MTT assays to evaluate cell viability. The results showed a significant decrease in cell viability following *CBX4* knockdown in both A549 and H1299 cells (Fig. 1B). Additionally, colony formation assays revealed suppressed cell growth subsequent to *CBX4* knockdown (Fig. 1C). Further EdU-incorporation assays were conducted, illustrating that *CBX4* knockdown impeded DNA replication, ultimately leading to the inhibition of cell proliferation (Fig. 1D).

To explore the effect of *CBX4* overexpression on LUAD cell proliferation, we transfected Flag-CBX4 expression constructs into A549 and H1299 cells (Fig. 1E). Subsequently, we performed MTT, colony formation, and EdU-incorporation assays. The results indicated that *CBX4* overexpression led to increased cell viability, a higher number of cell colonies, and enhanced DNA replication activity (Fig. 1F, H). Taken together, these findings collectively demonstrate that CBX4 promotes the proliferation of LUAD cells in vitro.

### CBX4 facilitates the growth of LUAD cells in vivo

**Fig. 2:**
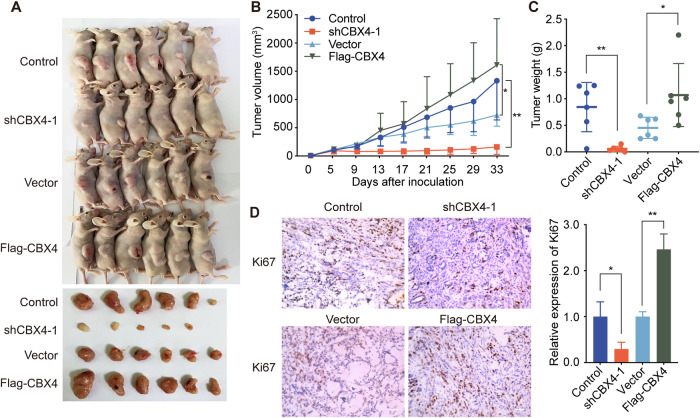
CBX4 facilitates the growth of LUAD in vivo. **A** A549 cells stably expressing control or *CBX4* shRNA, empty vectors or Flag-CBX4, were transplanted into female athymic nude mice. Tumors were stripped out 33 days later and photographed. **B** After implantation into mice, tumors were measured every 4 days using a Vernier caliper, and tumor volume was calculated using the formula: *V* = *π*/6 × length × width^2^. **C** The tumors were weighed. For figures **B**, **C**, each bar represents the mean ± SD for 6 animal measurements; *P < 0.05, **P < 0.01 (Student’s *t*-test). **D** Immunohistochemical staining of frozen sections was performed using Ki67 antibodies. Scale bar, 100 μm. The relative expression of Ki67 was calculated. Each bar represents the mean ± SD for *n* = 3, *P < 0.05, **P < 0.01 (Student’s *t*-test).

To further elucidate the proliferation-promoting role of CBX4 in LUAD in vivo, we utilized a mouse subcutaneous tumor-bearing model to assess the impact of *CBX4* knockdown and overexpression on LUAD growth. A549 cells stably expressing control shRNAs, *CBX4* shRNAs, empty vector, or Flag-CBX4 were subcutaneously injected into nude mice. Tumor size was monitored every four days starting from day 5 post cell injection. After 33 days of tumor transplantation, the mice were euthanized, and the tumors were dissected, weighed, and photographed (Fig. 2A). The results demonstrated a significant reduction in tumor volume and weight in the *CBX4* knockdown group compared to the control group (Fig. 2B, C). Conversely, a notable increase in tumor volume and weight was observed in the *CBX4* overexpression group compared to the empty vector group (Fig. 2B, C). Furthermore, tumors with *CBX4* knockdown exhibited lower malignancy compared to those formed by control A549 cells (Fig. 2D), while tumors with *CBX4* overexpression displayed higher malignancy than the empty vector group, as indicated by Ki67 staining (Fig. 2D). These data robustly support the assertion that CBX4 promotes LUAD cell growth in vivo.

### CBX4 inhibits LUAD cells metastasis in vitro and in mice

**Fig. 3:**
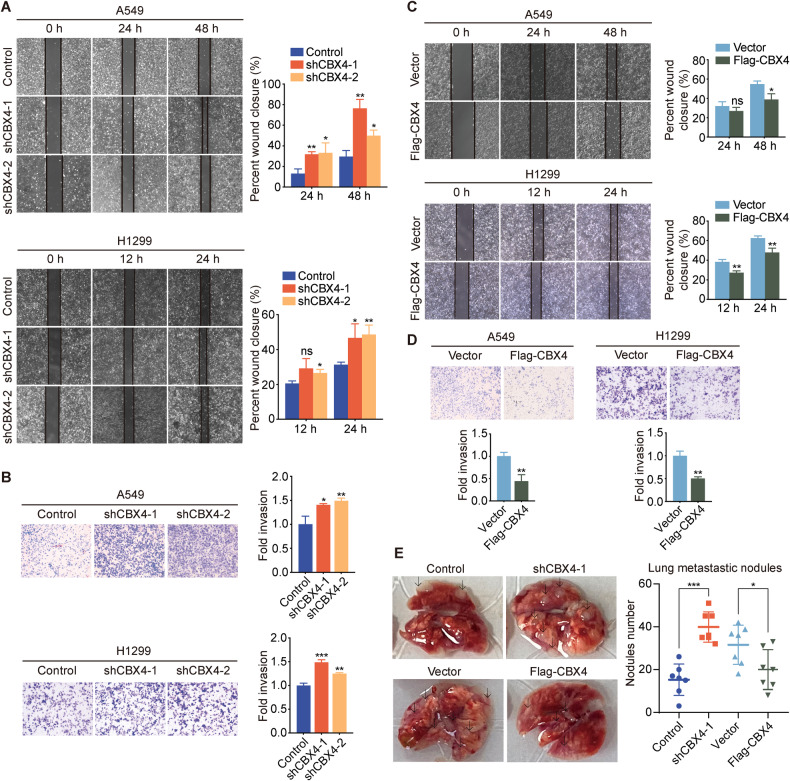
CBX4 suppresses the invasion and metastasis of LUAD cells in vitro and in vivo. **A** A549 and H1299 cells expressing control or *CBX4* shRNA were subjected to wound-healing assays. **B** Transwell assays were performed in A549 and H1299 cells expressing control or *CBX4* shRNA. Scale bars, 100 μm. **C** Wound-healing assays were performed using cells overexpressing empty vectors or Flag-CBX4. **D** Transwell assays were carried out using A549 and H1299 cells with or without *CBX4* overexpression. Scale bars, 100 μm. For figures **A**–**D**, each bar represents the mean ± SD for n = 3; ns: no significant, *P < 0.05, **P < 0.01, ***P < 0.001, indicating shCBX4 versus control, Flag-CBX4 versus vector (Student’s *t*-test). **E** A549 cells stably expressing control shRNAs, *CBX4* shRNAs, empty vector, or Flag-CBX4 were intravenously injected into the tail of female athymic nude mice. The mice were decapitated 8 weeks later, and the number of pulmonary metastatic nodules was counted, highlighted by arrows. Each bar represents the mean ± SD for 7 animal measurements; *P < 0.05, **P < 0.01, ***P < 0.001, indicating shCBX4-1 versus control, Flag-CBX4 versus vector (Student’s *t*-test).

In addition to cell proliferation, the migratory and invasive capabilities of tumor cells stand as critical indicators of malignancy. Therefore, we explored the influence of *CBX4* overexpression and knockdown on the migration and invasion of lung adenocarcinoma cells. Initially, we conducted wound-healing assays to assess cell migration. The results showed a remarkable increase in the migration of A549 and H1299 cells upon *CBX4* knockdown (Fig. 3A). Furthermore, the transwell assay illustrated that *CBX4* knockdown markedly enhanced the invasive potential of both A549 and H1299 cells (Fig. 3B). Conversely, consistent outcomes from the wound-healing and transwell assays revealed that *CBX4* overexpression attenuated the migratory and invasive abilities of lung adenocarcinoma cells (Fig. 3C, D). These results collectively demonstrate that CBX4 inhibits the migration and invasion of lung adenocarcinoma cells in vitro.

To evaluate the impact of *CBX4* knockdown and overexpression on lung adenocarcinoma cell metastasis in vivo, we injected A549 cells stably expressing control shRNAs, *CBX4* shRNAs, empty vector, or Flag-CBX4 into the tail veins of 4-5-week-old NOD/SCID mice. After 8 weeks, the mice were euthanized, and the lungs were dissected, photographed, and analyzed for tumor nodules. The results demonstrated a remarkable increase in the number of metastatic tumor nodules in the lungs of mice injected with *CBX4* knockdown A549 cells compared to the control group (Fig. 3E). Conversely, there was a significant reduction in the number of metastatic tumor nodules in the lungs of mice injected with *CBX4* overexpression A549 cells compared to the empty vector group (Fig. 3E). In conclusion, these findings suggest that CBX4 inhibits the metastasis of lung adenocarcinoma cells in mice, aligning with the observed phenotype in vitro.

### CBX4 regulates the transcription of genes related to cell proliferation and invasion

**Fig. 4:**
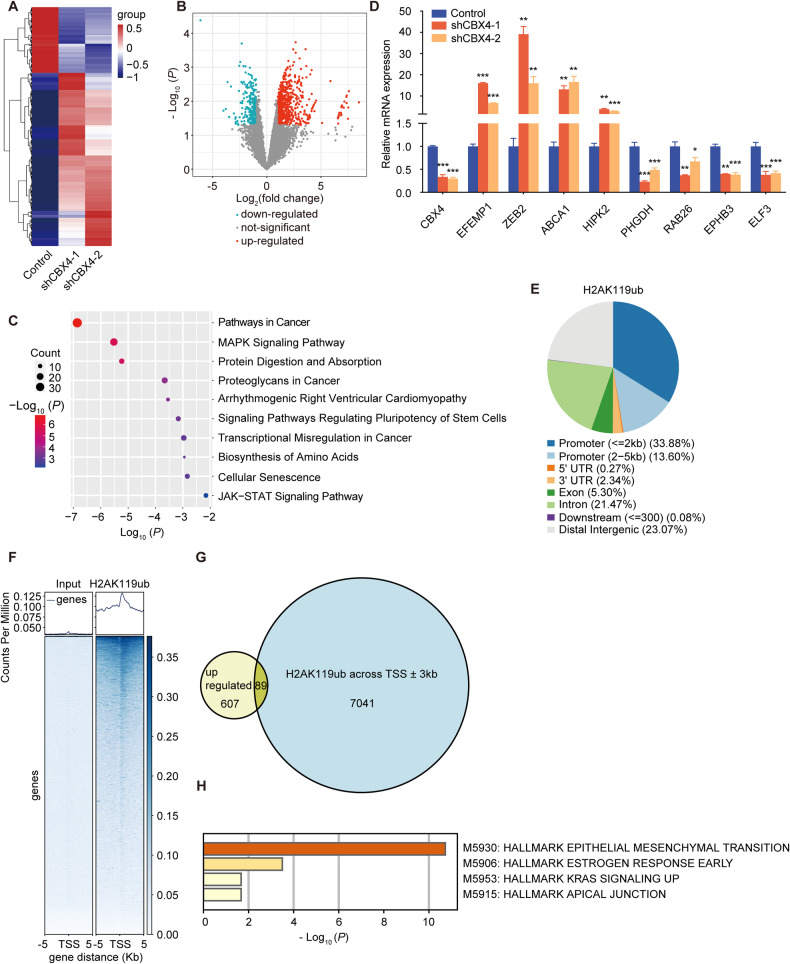
CBX4 regulates the transcription of genes related to cell proliferation and invasion. **A** The mRNAs from A549 cells expressing control or *CBX4* shRNAs were extracted and subjected to RNA-seq. The heatmaps show differentially expressed genes following *CBX4* knockdown. **B** The volcano plot shows the differentially expressed genes between A549 cells expressing control and *CBX4* shRNAs. Genes meeting the criteria of *P* < 0.05 and fold change ≥ 2 are depicted as red dots for upregulated genes and blue dots for downregulated genes. **C** KEGG pathway enrichment analyses of significantly dysregulated genes in *CBX4* knockdown A549 cells. **D** Total mRNA from A549 cells expressing indicated shRNAs was extracted and real-time quantitative RT-PCR assays were performed. Each bar represents the mean ± SD for *n* = 3; *P < 0.05, **P < 0.01, ***P < 0.001 versus control (Student’s *t*-test). **E** ChIP using H2AK119ub antibodies was carried out in A549 cells, and the eluted DNA was subjected to high-throughput sequencing. The pie chart visualizes the distribution of H2AK119ub across the genome. **F** The profile diagram and heatmap exhibit H2AK119ub signal levels and patterns across the 5 kb upstream of TSS to 5 kb downstream of TSS of genes. **G** The Venn diagram illustrates the overlap between genes modified by H2AK119ub at TSS ± 3 kb and those upregulated by *CBX4* knockdown obtained from RNA-seq data. **H** Hallmark pathway enrichment analyses were performed on the overlapping genes in figure G.

Building upon our prior observations regarding CBX4’s influence on the proliferation and migration of LUAD cells, we sought deeper insights into the underlying molecular mechanisms. To this end, we conducted RNA-seq analysis in *CBX4* knockdown and control A549 cells. The resulting heatmap unveiled significant alterations, indicating that *CBX4* knockdown led to the upregulation of 696 genes and the downregulation of 264 genes compared to the control group (Fig. 4A, B). Kyoto Encyclopedia of Genes and Genomes (KEGG) enrichment analysis of these differentially expressed genes underscored their marked involvement in “pathways in cancer” and “MAPK signaling pathway” (Fig. 4C).

Subsequently, we selected eight genes with substantial expression differences and conducted real-time quantitative RT-PCR experiments to validate their expression alteration in control and *CBX4* knockdown cells. The results consistently corroborated the RNA-seq findings. Notably, *EFEMP1*, *ZEB2*, *ABCA1*, and *HIPK2* were upregulated upon *CBX4* knockdown, while *PHGDH*, *RAB26*, *EPHB3*, and *ELF3* exhibited downregulation (Fig. 4D).

Previous studies have highlighted CBX4’s crucial role as a component of canonical PRC1 and its involvement in transcriptional repression [17, 18]. By utilizing anti-H2AK119ub, we performed ChIP-seq analysis in A549 cells, which revealed significant recruitment of H2AK119ub in gene promoter regions across the genome (Fig. 4E, F). Furthermore, we conducted an overlap analysis between genes upregulated due to *CBX4* knockdown in RNA-seq and those modified by H2AK119ub at TSS ± 3 kb as identified in ChIP-seq analysis, and discovered 89 genes (Fig. 4G). Pathway enrichment analysis of these 89 genes unveiled their significant involvement in the epithelial-mesenchymal transition signaling pathway (Fig. 4H). This suggests that CBX4 potentially inhibits the migration of LUAD cells through transcriptional repression of these downstream target genes.

### CBX4 inhibits the migration and invasion of LUAD cells by suppressing *ZEB2* transcription

**Fig. 5:**
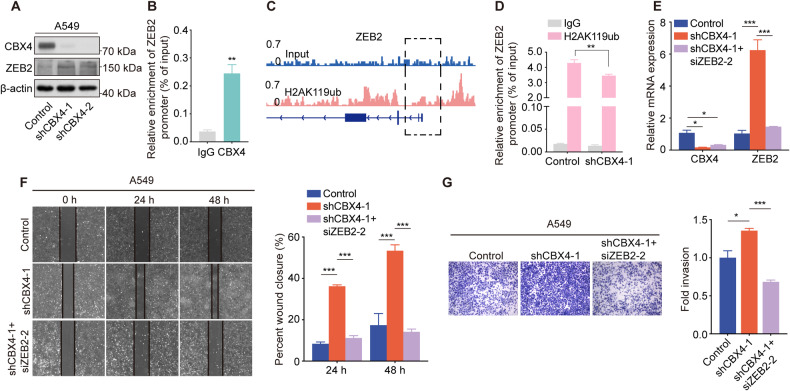
CBX4 inhibits the migration and invasion of LUAD cells by suppressing the transcription of *ZEB2.* **A** Western blot analysis of ZEB2, CBX4, and β-actin expression in A549 cells expressing control or *CBX4* shRNAs. **B** ChIP-qPCR assays were performed using anti-CBX4 or IgG, with primer pairs targeting the *ZEB2* promoter in A549 cells. **C** Genome browser view of the H2AK119ub signal on the *ZEB2* gene obtained by ChIP-seq in A549 cells. **D** ChIP-qPCR assays were performed using anti-H2AK119ub or IgG, along with primer pairs targeting the *ZEB2* promoter in A549 cells expressing control and shCBX4-1. For figures **B** and **D**, each bar represents the mean ± SD for *n* = 3; **P < 0.01 versus control (Student’s *t*-test). **E** A549 cells stably expressing control shRNA or *CBX4* shRNA-1 were transfected with *ZEB2* siRNAs or not. The mRNAs were extracted, and subjected to real-time quantitative RT-PCR assay. **F** Wound-healing assays were performed using indicated cells. **G** Transwell assays were performed using A549 cells expressing control shRNA, shCBX4-1, and cells expressing both shCBX4-1 and siZEB2-2. Scale bars, 100 μm. For figures E-G, each bar represents the mean ± SD for *n* = 3; *P < 0.05, ***P < 0.001 (one-way ANOVA followed by Bonferroni post-hoc test or Tambane’s T2 post-hoc test).

ZEB2, a DNA-binding transcriptional regulator, interacts with the E-box sequence on the *E-cadherin* promoter, leading to the downregulation of *E-cadherin* transcription [19]. Therefore, ZEB2 plays a crucial role in inducing epithelial-mesenchymal transition (EMT) [20, 21]. Through RNA-seq analysis and subsequent validation, we observed an upregulation of *ZEB2* transcription following *CBX4* knockdown (Fig. 4D). Western blot analysis confirmed an increase in ZEB2 protein level upon *CBX4* knockdown (Fig. 5A). These findings prompted us to investigate whether CBX4 inhibits LUAD cell migration via transcription repression of *ZEB2*.

To test this hypothesis, we performed chromatin immunoprecipitation (ChIP) assays in A549 cells using anti-CBX4 or control IgG, along with primers targeting the *ZEB2* promoter regions. The results revealed the occupancy of CBX4 on the promoter region of *ZEB2* (Fig. 5B). CBX4-PRC1 represses gene transcription by establishing H2AK119ub on the promoter of target genes [22]. ChIP-seq assays conducted in A549 cells revealed a prominent enrichment of H2AK119ub on the *ZEB2* promoter (Fig. 5C). Furthermore, the occupancy of H2AK119ub on the promoter of *ZEB2* was validated by ChIP-qPCR assays using H2AK119ub antibodies (Fig. 5D). Moreover, CBX4 depletion reduced the enrichment of H2AK119ub on the *ZEB2* promoter (Fig. 5D). These findings provide evidence that CBX4 downregulates the transcription of *ZEB2* by directly binding to its promoter to establish H2AK119ub.

To further prove that the promotion of cell migration caused by *CBX4* knockdown was mediated by the upregulation of *ZEB2*, we knocked down the expression of *ZEB2* by specific siRNAs, and detected its effect on the proliferation and migration of A549 cells. The efficiency of *ZEB2* knockdown by its specific siRNAs was examined by real-time quantitative RT-PCR assays (Fig. S1A). To further evaluate whether ZEB2 regulates the growth of A549 cells, MTT and EdU-incorporation assays were performed in control or *ZEB2* knockdown A549 cells. The results showed that the proliferation of A549 cells was unaffected by *ZEB2* knockdown (Fig. S1B, C). However, depletion of ZEB2 inhibited the migration and invasion of A549 cells (Figure S1D and S1E). Then, we transfected *ZEB2* siRNAs into *CBX4* knockdown cells (Fig. 5E) and evaluated cell migration and invasion using wound-healing and transwell assays. The results revealed that knockdown of *ZEB2* restored the promoted invasive ability of A549 cells caused by *CBX4* knockdown (Fig. 5F, G), proving that *ZEB2* is a functional target gene of CBX4 in suppressing LUAD metastasis.

### CBX4 promotes the proliferation of LUAD via transcriptional activation of *PHGDH*

**Fig. 6:**
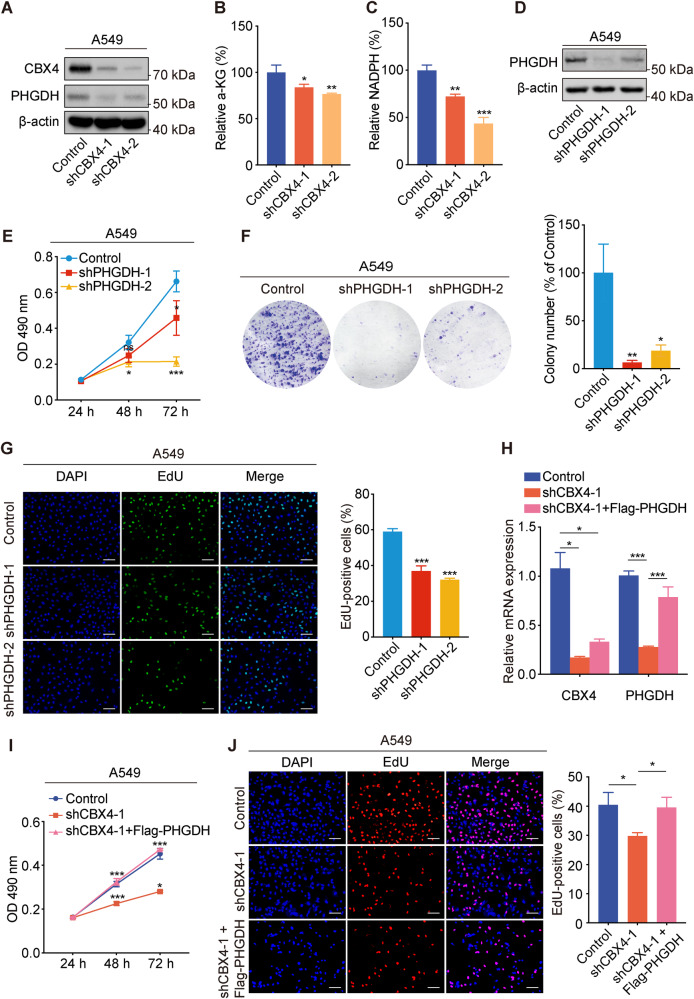
CBX4 promotes the proliferation of LUAD via transcriptional activation of *PHGDH.* **A** Western blot analysis of the expression of PHGDH, CBX4, and β-actin in A549 cells expressing control or *CBX4* shRNAs. The levels of a-KG (**B**) and NADPH (**C**) in the cell lysate from A549 cells expressing control or *CBX4* shRNAs were detected by HPLC and spectrophotometry, respectively. **D** Western blotting of cell lysates from A549 cells expressing control or *PHGDH* shRNAs, using PHGDH and β-actin antibodies. **E** MTT assays were performed in A549 cells expressing control or *PHGDH* shRNAs. **F** Colony formation assays were conducted in A549 cells expressing control or *PHGDH* shRNAs. The number of colonies was counted. **G** EdU-incorporation assays were performed in indicated cells, and the percentage of EdU-positive cells was calculated. Scale bar, 100 μm. For figures **B**, **C** and **E**–**G**, each bar represents the mean ± SD for *n* = 3; *P < 0.05, **P < 0.01, ***P < 0.001 versus control (Student’s *t*-test). **H** A549 cells stably expressing control shRNA or *CBX4* shRNA-1 were transfected with Flag-PHGDH expression constructs or empty vectors. The mRNAs were extracted, and subjected to real-time quantitative RT-PCR assays. **I** MTT assays were performed in the indicated cells. **J** EdU-incorporation assays were performed in the indicated cells. Scale bars, 100 μm. For figures **H**–**J**, each bar represents the mean ± SD for *n* = 3; *P < 0.05, ***P < 0.001 (one-way ANOVA followed by Bonferroni post-hoc test or Tambane’s T2 post-hoc test).

Understanding the regulatory mechanisms by which CBX4 influences LUAD proliferation remains elusive. Our RNA-seq analyses demonstrated that knockdown of *CBX4* leads to the downregulation of phosphoglycerate dehydrogenase (PHGDH) transcription (Fig. 4D). Subsequent Western blot analysis confirmed a reduction in PHGDH protein levels following *CBX4* knockdown (Fig. 6A). PHGDH, a key serine biosynthesis enzyme, participates in metabolic reprogramming. Serine is vital for the biosynthesis of numerous macromolecules necessary for cell proliferation. Cancer cells that overexpress PHGDH have a high inclination towards serine synthesis, leading to tumorigenesis through upregulation of serine biosynthesis pathways. PHGDH promotes tumorigenesis in many cancer types, such as pancreatic cancer [23], colorectal cancer [24], and liver cancer [25]. Serine biosynthesis pathway generates α-ketoglutarate as a byproduct of serine metabolism, and serine synthesis is crucial for producing NADPH in both the mitochondrial one-carbon and folate metabolic pathways [26, 27]. To investigate whether reduced PHGDH due to CBX4 depletion impacts serine biosynthesis, we measured cellular α-ketoglutarate and NADPH levels in control or *CBX4* knockdown cells. The results revealed decreased levels of α-ketoglutarate and NADPH upon *CBX4* knockdown, indicating the downregulation of serine biosynthesis by CBX4 depletion in LUAD cells (Fig. 6B, C).

To establish the relationship between the inhibition of cell proliferation caused by *CBX4* knockdown and the downregulation of *PHGDH*, we intended to first examine the effects of *PHGDH* knockdown on LUAD cell proliferation. The efficiency of *PHGDH* knockdown by its specific shRNAs was confirmed through Western blot analysis (Fig. 6D). Subsequent MTT assays demonstrated a remarkable decrease in cell viability upon *PHGDH* knockdown in A549 cells (Fig. 6E). Additionally, colony formation and EdU-incorporation assays revealed suppressed cell proliferation following *PHGDH* knockdown (Fig. 6F, G). On the other hand, we overexpressed Flag-PHGDH in A549 cells (Fig. S2A), and conducted MTT and EdU-incorporation assays. The results revealed enhanced proliferation upon *PHGDH* overexpression (Fig. S2B, C). Then, we overexpressed Flag-PHGDH in *CBX4* knockdown cells (Fig. 6H) and examined cell proliferation. The results of MTT and EdU-incorporation assays demonstrated that overexpression of *PHGDH* significantly reversed the reduction in cell proliferation induced by *CBX4* knockdown (Fig. 6I, J). These findings indicate that *CBX4* knockdown inhibits the proliferation of LUAD cells through the downregulation of *PHGDH*.

### CBX4 increases *PHGDH* transcription via recruiting GCN5 to the *PHGDH* promoter

**Fig. 7:**
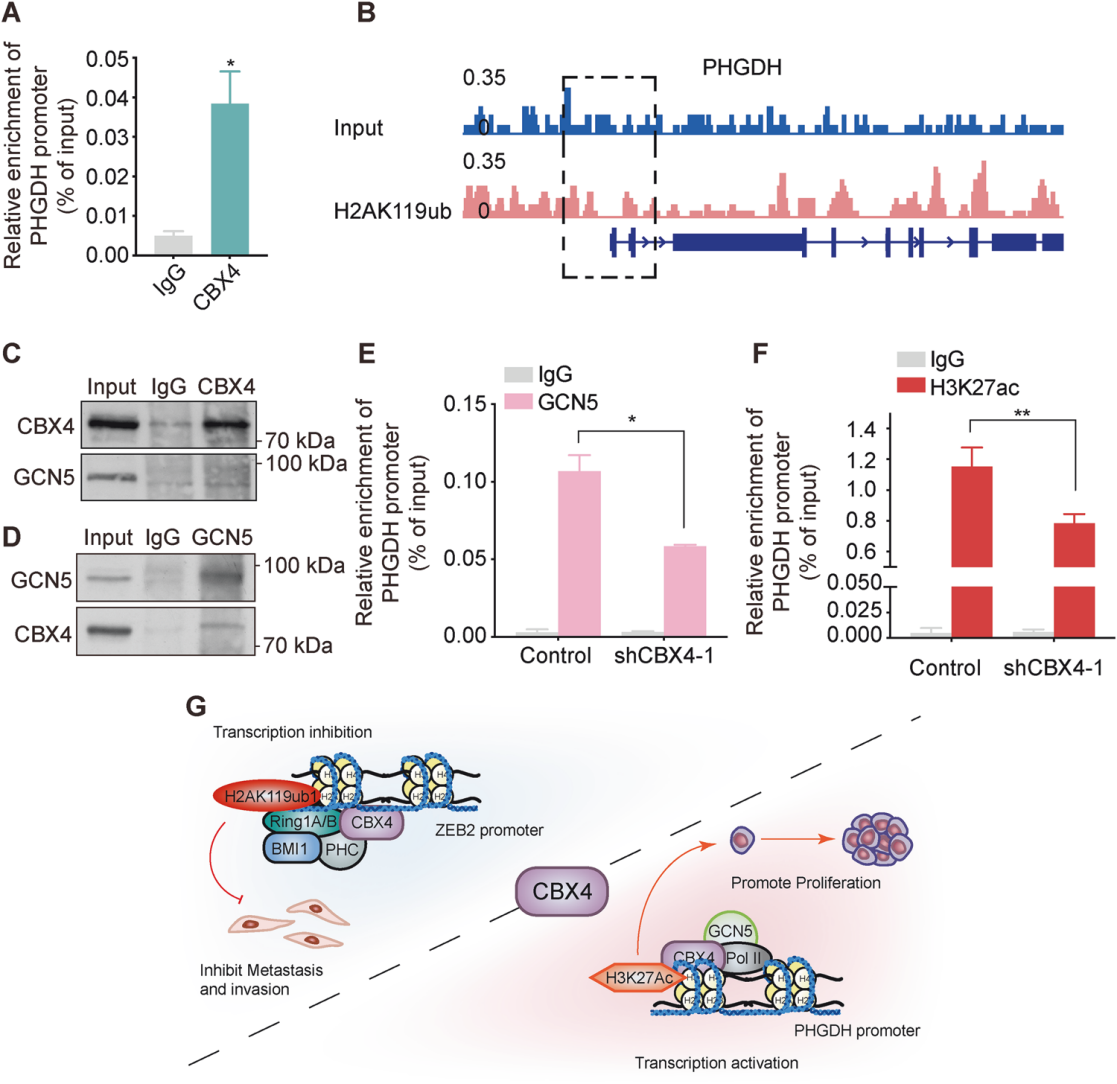
CBX4 increases *PHGDH* transcription via recruiting GCN5 to the *PHGDH* promoter. **A** ChIP-qPCR assays were performed using anti-CBX4 or IgG, with primer pairs targeting the *PHGDH* promoter in A549 cells. **B** Genome browser view of the H2AK119ub signal on the *PHGDH* gene obtained by ChIP-seq in A549 cells. **C**-**D** Immunoprecipitation assays were carried out with anti-CBX4 (**C**) or anti-GCN5 (**D**), followed by immunoblotting analysis in A549 cells. ChIP-qPCR was performed using anti-GCN5 (**E**) and anti-H3K27ac (**F**) with primer pairs targeting the *PHGDH* promoter in A549 cells expressing control shRNA and shCBX4-1. For figures **A**, **E**, and **F**, each bar represents the mean ± SD for *n* = 3; *P < 0.05, **P < 0.01 versus control (Student’s *t*-test). **G** The sketch map presents the dual role of CBX4 in transcriptional regulation and LUAD progression.

To ascertain whether CBX4 directly regulates the transcription of *PHGDH*, ChIP-qPCR experiments were conducted using anti-CBX4 or lgG, with primers targeting the *PHGDH* promoter. The results revealed a significant binding of CBX4 to the *PHGDH* promoter (Fig. 7A). Notably, ChIP-seq assays in A549 cells discovered no enrichment of H2AK119ub on the *PHGDG* promoter (Fig. 7B), further suggesting the involvement of alternative molecular mechanisms in CBX4-mediated transcriptional activation of *PHGDH*.

Previous studies have elucidated the interaction between CBX4 and GCN5 [28], a histone acetyltransferase known for its role as a transcriptional activator by acetylating specific histone residues, including H3K14 and H3K27 [29]. To validate this interaction in A549 cells, we performed endogenous co-immunoprecipitation assays. The results unequivocally confirmed the physical interaction between CBX4 and GCN5 in LUAD cells (Fig. 7C, D). Furthermore, following *CBX4* knockdown, the binding of GCN5 to the *PHGDH* promoter decreased (Fig. 7E), concomitant with a reduction in H3K27ac enrichment on the *PHGDH* promoter region (Fig. 7F). Collectively, these findings suggest that CBX4, functioning as a transcriptional activator, recruits GCN5 to the *PHGDH* promoter region, thereby facilitating the establishment of H3K27ac, which in turn facilitates *PHGDH* transcription.

## Discussion

This study identified CBX4 as an important regulator in the growth and metastasis of lung adenocarcinoma. Our data demonstrated that CBX4 plays a bidirectional role in LUAD progression, promoting proliferation while inhibiting metastasis. Mechanistically, CBX4 exerts transcriptional activation or inhibition by interacting with different epigenetic factors. CBX4 interacts with GCN5 to activate *PHGDH* transcription, promoting LUAD proliferation, whereas CBX4 participates in PRC1-mediated suppression of *ZEB2* transcription to suppress LUAD metastasis (Fig. 7G).

The CBX proteins, integral components of cPRC1, exert pivotal roles in directing cPRC1 to distinct genomic loci, maintaining repressive transcriptional states. However, the question of whether these CBX proteins recruit PRC1 to disparate targets and whether distinct CBX-PRC1 complexes hold varying biological functions warrants thorough investigation. Notably, these five CBX proteins display distinct expression patterns specific to haematopoietic cell stages, potentially forming diverse cPRC1 complexes during haematopoietic differentiation [30]. Similarly, their unique and nonoverlapping functions manifest in the pluripotency and differentiation of stem cells [31]. *CBX7* overexpression augments embryonic stem cell (ESC) self-renewal and impedes ESC differentiation, while elevated CBX2, CBX4, or CBX8 levels induce pluripotent stem cell differentiation [31]. However, the delineation of these proteins’ distinct roles in LUAD progression remains unexplored. Our previous study demonstrated their differential expression patterns in LUAD: upregulation of CBX2, CBX4, and CBX8 alongside downregulation of CBX6 and CBX7 [16]. Specifically, we identified CBX2-PRC1’s direct inhibition of several PPAR signaling pathway genes and tumor suppressors, fostering LUAD proliferation and metastasis [32]. Furthermore, CBX8-PRC1 promotes lung adenocarcinoma growth and metastasis through transcriptional repression of *CDKN2C* and *SCEL* [16]. In this study, we showed that, while CBX4 exhibits high expression in LUAD akin to CBX2 and CBX8, it exerts a bidirectional role in LUAD proliferation and metastasis. CBX4-GCN5 induces *PHGDH* transcription to promote LUAD proliferation, whereas CBX4-PRC1 impedes *ZEB2* expression to suppress LUAD metastasis (Fig. 7G). In fact, a better way to detect the similarities and differences in the distribution of five CBX proteins on the genome of lung adenocarcinoma cells is to conduct ChIP-seq experiments using antibodies against each of the five CBX proteins simultaneously. However, despite multiple attempts, only antibodies against CBX2 and CBX8 can be used for ChIP-seq. When better antibodies appear in the future, this experiment is still worth trying. In summary, our work further elucidates that different CBX proteins have different target genes in LUAD cells and perform different biological functions.

Distinguished from other CBX proteins within cPRC1, CBX4 exhibits dual roles in transcriptional regulation-either repression or activation depending on its context and interacting partners. As a component of cPRC1, the canonical role of CBX4 involves recognition of PRC2-mediated H3K27me3, facilitating the recruitment of PRC1 to the H3K27me3 mark, leading to subsequent transcriptional repression [18]. For instance, CBX4 suppresses transcription through binding to the promoters of target genes, such as *p16* [33] and *GATA4/GATA6* [34]. Additionally, CBX4 participates in transcriptional regulation through its SUMO E3 ligase activity. For example, CBX4 promotes HIF-1α SUMOylation at K391 and K477, thereby enhancing its DNA binding ability and transcriptional activity, ultimately augmenting VEGF expression [35]. CBX4 also catalyzes the SUMOylation of SIP1 to diminish its transcriptional repression of *E-cadherin* [36, 37]. Beyond its activities related to cPRC1 and SUMO E3 ligase function, CBX4’s involvement in transcriptional regulation extends to its association with other epigenetic factors. CBX4 can recruit GCN5 to the RUNX2 promoter, leading to upregulated RUNX2 expression in osteosarcoma [38]. Conversely, in colorectal carcinoma, CBX4 suppresses RUNX2 expression by recruiting HDAC3 to the Runx2 promoter [39]. In our study, we elucidated CBX4’s inhibition of *ZEB2* transcription through the recruitment of cPRC1 to establish H2K119ub on the *ZEB2* promoter. Simultaneously, CBX4 promoted *PHGDH* transcription by interacting with GCN5, augmenting histone acetylation on the *PHGDH* promoter (Fig. 7G). These findings underscore the diverse transcriptional regulatory functions of CBX4, contingent upon its interactions with distinct epigenetic factors. Under what conditions, CBX4 is more inclined to bind with GCN5 to exert transcriptional activation, and under what conditions, CBX4 is more present in the PRC1 complex to implement transcriptional inhibition, which was not explored in this study, and deserves further research in the future.

CBX4 has garnered extensive attention in cancer research, predominantly recognized as an oncogene due to its implications in tumor progression across various cancer types, including breast cancer [40], hepatocellular carcinoma [41], osteosarcoma [38], gastric cancer [42] and clear cell renal cell carcinoma [43]. Conversely, contrasting findings suggest a potential tumor-suppressive function for CBX4. In colorectal cancer, CBX4 suppresses metastasis by recruiting HDAC3 to deacetylate histone H3K27 at the *RUNX2* promoter, consequently inhibiting RUNX2 expression [39]. In our study, we unveiled the dichotomous role of CBX4 in the progression of lung adenocarcinoma, where it acts as a double-edged sword. CBX4 exhibits the ability to promote the proliferation of lung adenocarcinoma cells while concurrently inhibiting their migration and invasion. Due to this dual functionality of CBX4 in lung adenocarcinoma progression, the expression level of CBX4 can serve as a biomarker to monitor the progression of LUAD. Furthermore, understanding the nuanced interplay between CBX4 and epigenetic factors sheds light on potential therapeutic avenues in LUAD. As CBX4 suppresses LUAD metastasis through transcriptional inhibition of *ZEB2*, activating the expression of CBX4 together with blocking the interaction between CBX4 and GCN5 in lung adenocarcinoma cells can inhibit the metastasis of LUAD without affecting tumor cell proliferation. In another way, inhibition CBX4 expression together with depletion of ZEB2 can also suppress the growth of LUAD without activating metastasis.

## Materials and methods

### Cell and reagents

Human lung adenocarcinoma cells A549 and H1299 were purchased from the National Infrastructure of Cell Line Resource (Shanghai, China). A549 cells were cultured in Dulbecco’s modified Eagle’s medium (DMEM), and H1299 cells were cultured in Roswell Park Memorial Institute 1640 (RPMI1640) medium, both supplemented with 10% fetal bovine serum (FBS) (Biological Industries, Beit HaEmek, Israel) at 37 °C in a humidified atmosphere with 5% CO_2_. All cells were tested for Mycoplasma contamination every 2 months during culture as a routine test in cell laboratory using Mycoplasma Detection Kit (D101, Vazyme Biotech Co., Ltd) to ensure that the cells were Mycoplasma free. Antibodies used were purchased from the following sources: anti-FLAG (M2, F3165) from Merck KGaA (Darmstadt, Germany); anti‐β‐actin (AC038) from ABclonal Technology Co. (Wuhan, Hubei, China); anti‐CBX4 (30559) and anti‐Ki67 (9027) from Cell Signaling Technology (Danvers, MA, USA); anti-GCN5 antibodies (ab217876), anti-PHGDH (ab57030), and anti-H3K27ac (ab4729) from Abcam Inc. (Cambridge, MA, USA); anti-H2AK119ub (PTM-1121) from PTM Bio (Hangzhou, China); anti-ZEB2 (SC-271984) and horseradish peroxidase (HRP)‐conjugated secondary antibodies (sc‐2030 and sc‐2031) from Santa Cruz Biotechnology (Santa Cruz, CA, USA).

### RNA interference and plasmids transfection

Small interfering RNA (siRNA) targeting *ZEB2* (siZEB2-1: GGAGACAGAUCAGUAAUAUUU, siZEB2-2: CCCUAUCAGUGUGAUAAAUUU) or a negative control siRNA (UUCUCCGAACGUGUCACGUTT) were transfected into cells using Lipofectamine RNAiMAX Transfection Reagent (13778150, Thermo Fisher Scientific Inc.) following the manufacturer’s guidelines. For lentivirus production, two effective *CBX*4 siRNA sequences (siCBX4‐1: GCCCUUCUUUGGGAAUAUAAU, siCBX4‐2: CGUGAUCGUGAUGAGCAAAUA), two effective PHGDH siRNA sequences (siPHGDH‐1: GCUUCGAUGAAGGACGGCAAA, siPHGDH‐2: CGCAGAACUCACUUGUGGAAU), or control siRNA sequences (CCUAAGGUUAAGUCGCCCUCG), were cloned into the pLKO.1 lentiviral shRNA vector for shRNA-mediated gene silencing. The full‐length coding sequence of CBX4 fused with a FLAG tag coding sequence at the N-terminal was cloned into a pCDH-CMV-MCS-EF1-Puro vector. Flag-PHGDH lentiviral expression plasmid was obtained from Prof. Qiujing Yu from Tianjin Medical University. These lentiviral constructs were then transfected into HEK293T cells along with packaging plasmids (PAX2, pMD2.G) using PEI (23966; Polyscience, PA, USA). Infectious lentiviruses were gathered and filtered through a 0.45 μm pore size filter at 24 and 48 h after transfection. A549 and H1299 cells were infected with the lentiviruses in the presence of polybrene (1 μg/mL), and puromycin (1.5 μg/mL) was added to select stable knockdown/overexpression cell lines.

### MTT assay

The effect of CBX4 depletion, *CBX4* overexpression, *PHGDH* knockdown, *PHGDH* overexpression, *CBX4* knockdown together with *PHGDH* overexpression, or *ZEB2* knockdown on cell viability was assessed via the 3-(4,5-dimethylthiazol-2-yl)-2,5-diphenyl-2H-tetrazolium bromide (MTT) assay. Briefly, cells (2000 per well) in the control or experimental group were seeded into 96-well plates and incubated for 24, 48, and 72 h. Subsequently, the medium was replaced with a 10% MTT solution (0.5 mg/mL MTT in serum-free DMEM) and incubated at 37 °C for 4 h. Afterward, cells were lysed using 110 μL of dimethyl sulfoxide (DMSO) for 30 min at room temperature, and absorbance was measured at 490 nm using a microplate reader. Three biological replicates were performed for each experiment.

### EdU-incorporation assay

The effect of CBX4 depletion, *CBX4* overexpression, *PHGDH* knockdown, *PHGDH* overexpression, *CBX4* knockdown together with *PHGDH* overexpression, or *ZEB2* knockdown on cell proliferation was detected using the EdU-incorporation assay performed according to the manufacturer’s guidelines. Briefly, cells in the control or experimental group were initially seeded at a density of 6000 cells per well in 96-well plates. After a 24-h incubation, the culture medium was replaced with an EdU-containing medium for 2 h, and the assay was conducted using BeyoClick™ EdU Cell Proliferation Kit with Alexa Fluor 594 or Alexa Fluor 488 (C0078S or C0071S, Beyotime, Shanghai, China). Cells were then fixed with 4% paraformaldehyde for 15 min. After rinsed three times with washing buffer (3% BSA in PBS), cells were permeabilized with 0.3% TritonX-100 in PBS for another 10 min, and then incubated with 100 μL of click reaction solution for 30 min at room temperature. After three times washes, cells were incubated with Hoechst 33342 for 10 min in the dark. Random fluorescent images were captured using a fluorescent microscope to ensure objectivity in data collection, followed by the calculation of the proportion of cells displaying EdU incorporation. Three biological replicates were performed for each experiment.

### Colony formation assay

To illustrate the effect of CBX4 depletion, *CBX4* overexpression, or *PHGDH* knockdown on cell growth, colony formation assays were performed. Briefly, 600 cells were seeded into individual wells of a 6-well plate, with the medium refreshed every three days. After a 14-day incubation, cells were rinsed twice with phosphate-buffered saline (PBS), fixed using a 4% paraformaldehyde solution for 15 min, stained with a 0.1% crystal violet solution (Solarbio, Beijing, China) for 15 min, and then photographed. Three biological replicates were performed for each experiment.

### Wound-healing assay

To examine the effect of CBX4 depletion, *CBX4* overexpression, *ZEB2* knockdown, or *CBX4* knockdown together with *ZEB2* knockdown on cell migration ability, wound-healing assays were performed. Cells in the control or experimental group were cultured in 6-well plates until reaching 100% confluence. A pipette tip was used to create a wound line across the cell monolayer. After three washes with PBS, cells were maintained in an FBS-free medium to halt proliferation. Imaging was performed using a light microscope at specified intervals, and the wound width was quantified using ImageJ software. Three biological replicates were performed for each experiment.

### Transwell assay

To detect the effect of CBX4 depletion, *CBX4* overexpression, *ZEB2* knockdown, or *CBX4* knockdown together with *ZEB2* knockdown on cell invasive ability, transwell assays were executed. Approximately 5 × 10^4^ cells were suspended in 500 μL of serum-free medium and placed into the upper chamber precoated with 100 μL of Matrigel (BD Biosciences) solution (8 μL Matrigel mixed with 92 μL medium). The lower chamber was filled with 600 μL of culture medium containing 10% FBS. Following a 24-h incubation, invaded cells on the bottom surface of the membrane were fixed using a 4% paraformaldehyde solution and stained with 0.1% crystal violet. Imaging was performed under a microscope, and cell counts were conducted. Three biological replicates were performed for each experiment.

### Co-immunoprecipitation (Co-IP)

Endogenous co-immunoprecipitation assays were performed to detect the interaction between CBX4 and GCN5. Cells were harvested using cell lysis buffer (50 mM Tris-HCl pH 7.4, 150 mM NaCl, 1 mM EDTA, and 0.5% Triton X-100) supplemented with protease inhibitor. The cell lysate was incubated with specific primary antibodies or normal IgG overnight. Protein A/G magnetic beads were added into the protein–antibody complex for incubation at 4 °C for 2 h. After rinsed three times with washing buffer (50 mM Tris-HCl pH 7.4, 150 mM NaCl, 1 mM EDTA, and 0.1% Triton X-100), proteins were eluted by denaturation with 1× loading buffer at 100 °C for 10 min. Then, the obtained supernatant was subjected to Western blotting.

### RNA isolation and RT-qPCR

To detect the effect of CBX4 depletion, *ZEB2* knockdown, or CBX4 depletion together with *ZEB2* knockdown on the transcription of target genes, RT-qPCR assays were performed. Total RNA was extracted from cells in the control or experimental group using TRIzol (P118, GenStar, Beijing, China) following the manufacturer’s instructions. For cDNA synthesis, 1 μg of total RNA was reverse transcribed using Hifair^TM^ III 1st Strand cDNA Synthesis SuperMix (11141ES60, Yeasen, Shanghai, China). RT-qPCR was performed using RealStar Power SYBR qPCR Mix (A311, GenStar). The relative mRNA expression was calculated using the 2^−ΔΔCt^ method. Three biological replicates were performed for each experiment. The primers used were shown in Supplementary Table 1.

### Chromatin immunoprecipitation (ChIP)

ChIP-seq was performed to investigate the distribution of H2AK119ub across the genome, and ChIP-qPCR was performed to detect the enrichment of histone modifications such as H2AK119ub and H3K27ac, or the recruitment of CBX4 and GCN5, on the promoter of target genes. Briefly, cells were washed twice with PBS and chemically cross-linked with 1% formaldehyde for 10 min at room temperature. The cross-linking reaction was subsequently quenched with 0.125 mol/L glycine. Cells were then harvested and resuspended in SDS buffer (50 mM Tris-HCl pH 8.0, 100 mM NaCl, 5 mM EDTA, and 0.5% SDS) supplemented with protease inhibitors. The resulting cell pellets were collected via centrifugation at 1200 rpm for 10 min, followed by resuspension in ice-cold immunoprecipitation (IP) buffer comprising 100 mM NaCl, 66.67 mM Tris-HCl (pH 8.0), 5 mM EDTA (pH 8.0), 0.33% SDS, and 1.67% Triton X-100. This suspension was then subjected to sonication, and the supernatant post-centrifugation was incubated overnight at 4 °C with specific antibodies targeting CBX4, H2AK119ub, GCN5, H3K27ac, or nonspecific IgG. Protein A/G beads (Bimake, Houston, TX, USA) were then added at 4 °C for 4 h. The beads were thoroughly washed three times with wash buffer 1 (150 mM NaCl, 0.1% SDS, 1% Triton X-100, 2 mM EDTA, and 20 mM Tris-HCl, pH 8.0) and once with wash buffer 2 (1% Triton X-100, 500 mM NaCl, 0.1% SDS, 2 mM EDTA, and 20 mM Tris-HCl, pH 8.0). To reverse the cross-linking, samples were incubated at 65 °C for 6 h, and subsequently, DNA was extracted for deep sequencing (Novgene Co., Ltd, Tianjin, China) or real-time quantitative PCR reactions. For ChIP-qPCR assays, three biological replicates were performed for each experiment. Primer sequences used for ChIP-qPCR were as follows: *ZEB2*-F, 5′-AGAATGTGCCTGACCCATGT-3′, *ZEB2*-R, 5′-GGGTGGGGGTGGTTAATAGC-3′ (located in −3 kb ~ −2 kb in the *ZEB2* promoter); *PHGDH*-F, 5′-TGCATCAGCTAGTCAGCGTA-3′, *PHGDH*-R, 5′-TCTGAGGTTGCCAAATCCC-3′ (located in -1 kb ~ TSS in the *PHGDH* promoter).

### Mouse xenograft models

To test the effect of *CBX4* knockdown or overexpression on the growth and metastasis of LUAD, mouse tumor-bearing experiments were performed. After 1 week of adaptive feeding, the mice were randomly divided. For the in vivo subcutaneous tumor model, 4 × 10^6^ A549 cells stably expressing control or *CBX4* shRNA, empty vectors or FLAG-CBX4, were subcutaneously injected into female athymic nude mice (BALB/c, Charles River) aged between 5 and 6 weeks, with 6 mice per group. Tumor size was measured every four days using a Vernier caliper, and the volume was calculated using the formula: *V* = *π*/6 × length × width^2^. The investigator was blinded to the group allocation of the animals during the experiment. On the 33rd day post-injection, the mice were euthanized, and subcutaneous tumors were isolated and photographed.

For the in vivo lung metastatic model, 1 × 10^6^ cells in PBS were injected into the tail vein of NOD/SCID mice (Charles River) aged 4-5 weeks, with 7 mice per group. After 8 weeks, the mice were euthanized, and the chest cavity was opened for counting and photographing tumor nodules.

### Immunohistochemistry

Tumors grafted into mice were surgically excised, embedded in Optimal Cutting Temperature Compound (OCT, #4583, SAKURA, Tokyo, Japan), and solidified at −80 °C to create frozen tissue sections. The sections, sliced into 8 μm thickness, were fixed in 4% paraformaldehyde for 5 min, blocked with 3% hydrogen peroxide for 10 min, and subsequently incubated in 10% goat serum for 1 h. Slides were further incubated with primary antibodies overnight at 4 °C, followed by incubation with secondary antibodies conjugated to HRP. Color development was achieved using a DAB substrate kit (ZLI-9017, ZSGB-BIO, Beijing, China), followed by counterstaining with hematoxylin. Imaging was conducted using an Olympus microscope.

### RNA-seq and ChIP-seq analyses

A549 cells expressing control, shCBX4-1, or shCBX4-2 were lysed using TRIzol reagent, and the resulting samples were sent to Annoroad Gene Technology for further RNA purification and subsequent RNA-seq. RNA-seq analysis procedure involving alignment and assembly, quantification, normalization, and differential expression analysis was performed using the HISAT2-StringTie-Deseq2 pipeline.

Following ChIP-seq, clean data were obtained by removing reads containing adapter, poly-N, and low-quality reads from raw data. Subsequent analyses were based on high-quality clean data. The reference genome (UCSC hg38) was utilized for mapping clean data to the human genome using Bowtie2 (v 2.4.5) with specific parameters: --no-mixed --no-discordant --no-unal. Mapped reads with high confidence were sorted, indexed, and transformed to bam format using SAMtools (v1.6). Removal of duplicates was carried out using Sambamba (v0.8.2). The bigwig files were calculated and normalized with counts per million (CPM) using bamCompare from Deeptools (v3.5.1). Signal visualization around TSS was generated by the computeMatrix function. Macs2 (v2.2.7.1) was employed for peak calling, followed by the removal of the blacklist (https://github.com/BoyleLab/Blacklist/) from all peaks through Bedtools (v2.30.0). Peak annotations were performed using ChIPseeker (v1.30.3).

### Statistical analyses

Data for each group were presented as mean ± standard deviation and analyzed using IBM SPSS statistical software (version 19.0). Comparisons between two groups were analyzed by Student’s *t*-test. Multiple group comparisons were conducted using one-way ANOVA followed by Bonferroni post-hoc test or Tambane’s T2 post-hoc test to examine statistical variances.

### Supplementary information

Supplementary figures and table

original western blot

## Data Availability

The raw and processed high-throughput sequencing data (RNA-seq and ChIP-seq) were deposited in the Gene Expression Omnibus (GEO) database under accession number GSE252231.
