# Supplementary material for: CBX4 plays a bidirectional role in transcriptional regulation and lung adenocarcinoma progression
Source: Cell Death Dis. 2024 May 30;15(5):378. doi: 10.1038/s41419-024-06745-z (PMC11140001; doi:10.1038/s41419-024-06745-z)
Supplement: Supplementary file 1 — Supplementary figures and table [file 41419_2024_6745_MOESM1_ESM.pdf]

Supplementary Table 1. Sequences of primers for qRT-PCR.

Gene Name	Forward (5’ - 3’)	Reverse (5’ - 3’)
CBX4	GCTGCTGATCGCCTTCCAGAAC	TTGGAACGACGGGCAAAGGTAG
ELF3	CATGACCTACGAGAAGCTGAGC	GACTCTGGAGAACCTCTTCCTC
EPHB3	AAGCAGCGACACGGCTCTGATT	GACACGTCGATCTCCTTGGCAA
HIPK2	AGCGTCATCACCATCAGCAGTG	AGTCGTGGACTGTGACACAGCT
RAB26	GCCTCCTTTGACAACATCCAGG	TCCCTCTTCACCACACGCTCAT
EFEMP1	GACGCACAACTGTAGAGCAGAC	GAGCCTGGTGTATTCACGCATC
BNC2	ACTCTGCGGGACTATGTCCGAG	ACCGCAGAAACTGCTGAAGGGT
ZEB2	AATGCACAGAGTGTGGCAAGGC	CTGCTGATGTGCGAACTGTAGG
PHGDH	CTTACCAGTGCCTTCTCTCCAC	GCTTAGGCAGTTCCCAGCATTC
ABCA1	CAGGCTACTACCTGACCTTGGT	CTGCTCTGAGAAACACTGTCCTC
ZEB2	AGAATGTGCCTGACCCATGT	GGGTGGGGGTGGTTAATAGC
PHGDH	TGCATCAGCTAGTCAGCGTA	TCTGAGGTTGCCAAATCCC

Supplementary Figures

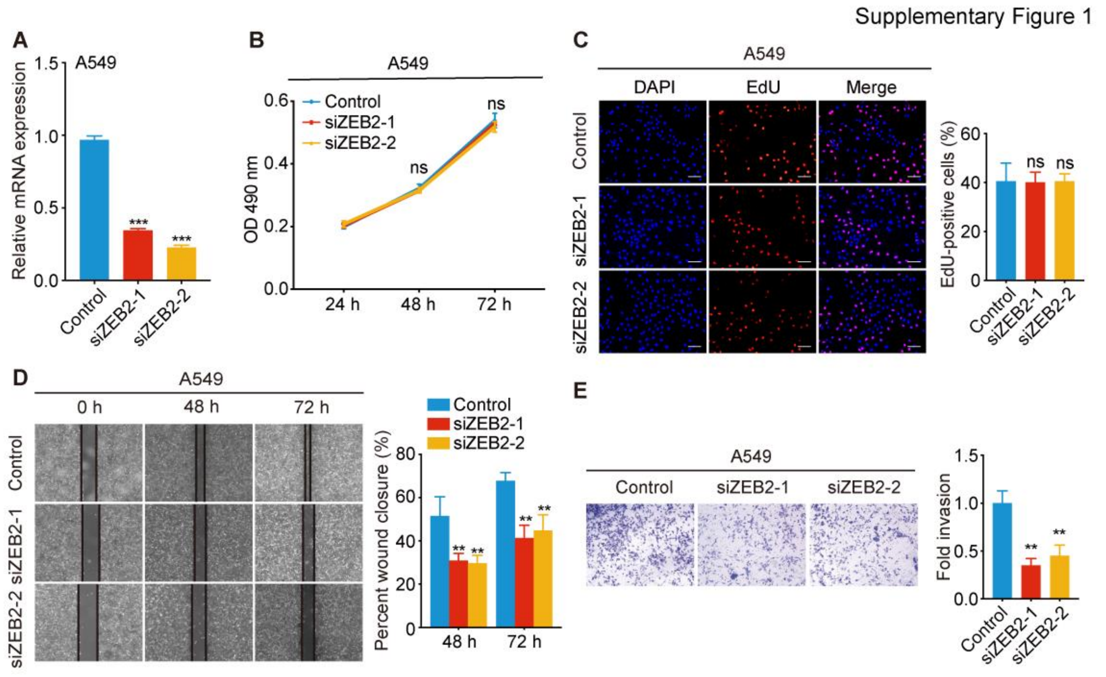

Supplementary Figure 1. *ZEB2* knockdown inhibits the invasion of LUAD cells without affecting cell proliferation. (A) The mRNAs from A549 cells transfected with control or *ZEB2* siRNAs were extracted and subjected to real-time quantitative RT-PCR assay. (B-C) MTT (B) and EdU-incorporation assays (C) were performed in A549 cells transfected with control or *ZEB2* siRNAs. Scale bars, 100 μm. (D-E) Wound-healing (D) and Transwell assays (E) were conducted in A549 cells transfected with control or *ZEB2* siRNAs. Scale bars, 100 μm. For figures A-E, each bar represents the mean ± SD for n = 3; ns: no significant, **P < 0.01, ***P < 0.001 versus control (Student's t-test).

Supplementary Figure 2

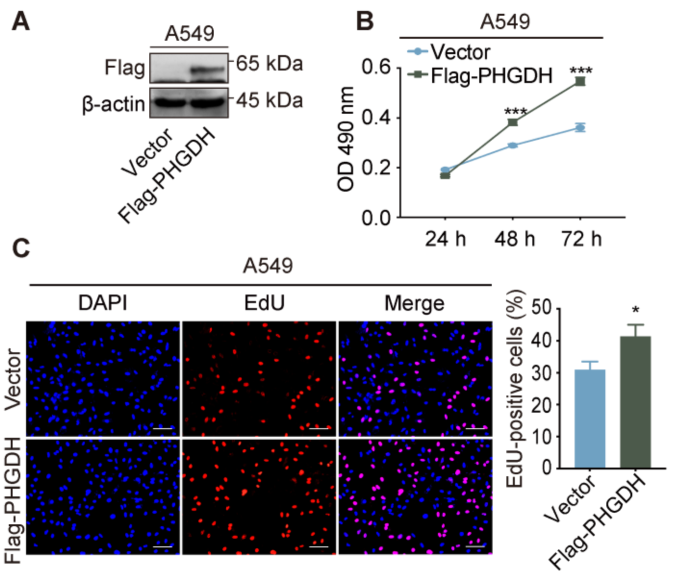

Supplementary Figure 2. PHGDH overexpression promotes the proliferation of LUAD cells. (A) The expression of Flag-PHGDH in A549 cells was detected by Western blotting. (B-C) MTT (B) and EdU-incorporation assays (C) were carried out in A549 cells overexpressing empty vectors or Flag-CBX4. Scale bars, 100 μm. For figures B-C, each bar represents the mean ± SD for n = 3; $*P < 0.05$, $***P < 0.001$ versus control (Student's t-test).
