# Supplementary figures and images for: CBX4 plays a bidirectional role in transcriptional regulation and lung adenocarcinoma progression
Source: Cell Death Dis. 2024 May 30;15(5):378. doi: 10.1038/s41419-024-06745-z (PMC11140001; doi:10.1038/s41419-024-06745-z)

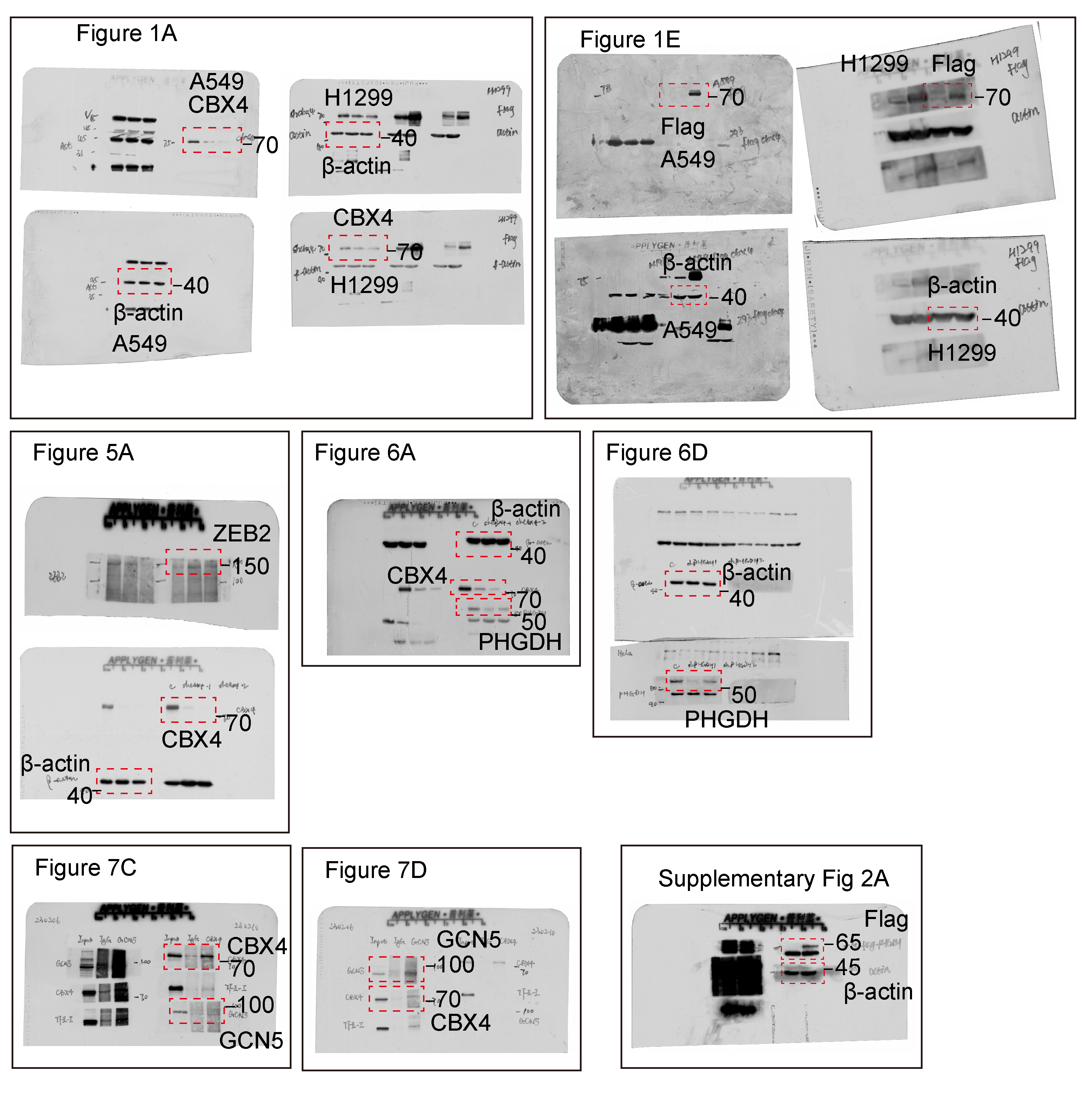

Supplement: Supplementary file 2 — original western blot [file 41419_2024_6745_MOESM2_ESM.tif]
